# The New York Institute of Stomatology

**Published:** 1905-05

**Authors:** 

**Affiliations:** The New York Institute of Stomatology


### Reports of Society Meetings.

### THE NEW YORK INSTITUTE OF STOMATOLOGY.

A MEETING of the Institute was held at the offices of Drs. Kimball and Parker, No. 27 West Thirty-eighth Street, New York, on Tuesday evening, January 2, 1905, the President, Dr. C. O. Kimball, in the chair.

The minutes of the last meeting were read and approved.

Dr. H. W. Gillett presented a useful appliance for the purpose of holding the gum back for cervical work, and also some appliances for holding the rubber dam for the same kind of cavities.

Dr. G. V. I. Brown, of Milwaukee, read a paper entitled "The Vital Significance of Oral Disease."

(For Dr. Brown's paper, see page 281.)

OK

DISCUSSION.

*Dr. H. L. Wheeler.*—In the first case mentioned by Dr. Brown he spoke of a necrotic condition, the patient dying of a general breaking up of the tissues. I would like to ask the essayist what the real lesion here was.

*Dr. Brown.*—It would be difficult to give an exact name to the trouble. Although the tissues were necrotic, there were no pus corpuscles. It was not in the nature of a malignant growth.

*Dr. Wheeler.*—A point that has been impressed upon my mind is that there seems to be a borderland uncultivated by any one, between where the physician or surgeon leaves off and where the dentist begins and as a result the patient is apt to suffer serious inconvenience if not grave dangers. I think, as a profession, we ought to pay more attention to these things that Dr. Brown has come here to show us to-night, and it has also sometimes seemed to me that physicians ought to pay more attention to the conditions of the mouth. I have myself, within a short space of time, had two cases where I was called in by a physician, and in each case a specialist had apparently been completely baffled. One case was thought to be antrum trouble, and was so treated without success. I found upon examination that it was an alveolar abscess, together with a curious condition of pain between the lateral and the bicuspid on the same side. There was a constant flow of pus coming from a sinus in the mucous membrane, back as far as the molars. From the history of the case this had been going on for a year or two, at intervals. The patient had been going to a nose and throat specialist constantly during that time. Another case came in recently with a bad swelling of the face, with a bright flush about the eye, together with a slight swelling and trouble in the mucous membrane of the mouth. It was an abscess on the palatal root of the first molar.

*Dr. Leo. Green.*—I should like to hear from Dr. Brown a little further regarding the case of noma.

*Dr. Brown.*—The case was referred to me by a physician, he having treated the patient for a week or two. When I first saw him the temperature was 104°, with an occasional rise to 105°, and it was only with a great amount of care that I was able to keep the temperature down. The original point of trouble appeared to be back of the third lower molar on the right side. The discharge was inky black and the tissue that died was black. An area very quickly formed in the cheek, and rapidly sloughed through. It was my custom, at each dressing, three or four times a day, to wipe off or remove with instruments the dead tissue, but the adjoining healthy tissue kept dying so rapidly that in two or three hours gangrenous masses again formed. The patient finally died in about ten days, being perfectly clear mentally until the last forty-eight hours. His last stage was marked by expectoration of red blood, the condition having extended into the lungs. There was no history of infection. The man was a laborer, and had apparently been in good health previously. There was, so far as I could find out, no history of syphilis, and the symptoms did not resemble syphilis in any way.

*Dr. C. F. Allan.*—The subjects dealt with this evening by the essayist are of very great importance to us in our work, in that they prove to us the correctness of our recent theories regarding the extraction of teeth. The essayist has shown us that the malformation of one or both jaws, whether caused by the extraction of a tooth or from other causes, affects the whole physical condition of the face and that a defective nasal septum or diseased nasal sinuses can sometimes be effectively treated by a widening of the upper arch. It is evident from this that, in performing these operations of expanding the arches, we are not only benefiting the patient in so far as their teeth are concerned, but we are also helping their breathing apparatus and benefiting the general conditions dependent upon free nasal passages and an abundant supply of oxygen. Care of these patients in early life would eliminate many of the nasal cases that come to the surgeon later on.

Dr. Bogue, who is not with us to-night, but to whom Dr. Brown has paid tribute, has for several years been paying close attention to the mouths of his very young patients, and he has asked many of us to send him models of such cases. He feels that there is a great advantage in the early correction of these deformities of the jaws, and it would now seem from Dr. Brown's paper that this early correction would greatly benefit some of the unfortunate conditions just described.

*Dr. Gillett.*—There are two points upon which I wish to say a word,—first, with regard to our duty toward those of our profession who have achieved the skill needful to such results as those shown us by Dr. Brown in the partially occupied field to which Dr. Wheeler has referred; if we cannot be active in this field we may at least direct the attention of the younger men toward it.

My second point pertains to the frequent neglect by practitioners of the chronic inflammations of the mouth and their sequelæ,—in particular, chronic alveolar abscess. It is not an uncommon thing to be told that some dentist has advised against "meddling" with such conditions. Such advice I believe to be malpractice, and that practitioners giving it should be held legally responsible for the ill results which frequently follow.

*Dr. J. B. Locherty.*—I would like to ask Dr. Brown at what age he operates in these cases of cleft palate.

*Dr. Brown.*—That depends entirely on the individual case. Always, if possible, before the child begins to speak; certainly before the speech changes from baby-talk to the talk of a grown person. I always attempt to build the child up as thoroughly as possible before operation.

*The President.*—In widening the arch in these cases, do you use a plate, or is it done by direct pressure upon the bones?

*Dr. Brown.*—I use an appliance attached to the teeth, where there are teeth, and a screw attached quite close to the gingival margin. I apply the force as rapidly as required, thus making direct pressure. The appliance does not attach to the central incisors. They are left free; therefore separation in this region is an indication of widening of the median suture. I sometimes have to spread the lower arch also, although it frequently follows the upper in course of development without immediate operative assistance.

*Dr. Allan.*—It has been stated that unless the central incisors are spread there can be no spreading of the bone.

*Dr. Brown.*—I do not see how that can be possible. Certainly I have accomplished this result without attaching my appliance to the central incisors.

*The President.*—In a case of cleft palate, the child being strong and the case favorable, how early would you operate?

*Dr. Brown.*—I doubt if any child ought to be considered strong enough for such an operation without danger earlier than one and one-half or two years of age. My preference would be older than this, yet at a sufficiently early age to prevent the fixation of incorrect speech habits. Naturally the conditions that modify and govern the surgeon's decisions as to time of operation in each individual case vary very greatly. Differences of health, ability to take nourishment, possibility of good post-operative care, and other important considerations have to be taken into account. Sometimes infants are brought long distances, with expectation of immediate operation, and we find that there may never be such opportunity for the child again, and so it becomes imperative to do the best that may be under the circumstances, but, if given a choice, there can be no question of the fact that more perfect results, both from a cosmetic point of view and that of capability of speech mechanism, can be obtained with less danger to the child by careful pre-operative care and treatment, and choosing the time for operation with full understanding of the benefit of willing co-operation in the post-operative treatment, which of course is impossible with very young infants.

*The President.*—I want to say just a word in regard to a case that came to my knowledge several years ago, relating to the borderline between the profession of the physician and the dentist. It is that of a lady nearly fifty years of age, who during an interval between her annual visits developed a sore on the outside of her face over the lower bicuspid. There was no trouble in the bicuspids nor the first two molars; the third molar, though only partially developed, was apparently in a healthy condition. I could find no connection between the little open sore and the wisdom-tooth. Her physician assured her that it was merely a local trouble, and had nothing whatever to do with the teeth. After it had gone on for a year or more I became convinced that that third molar had something to do with it, and had it removed, finding it dead. Even then I could find no connection between it and the sore, though the latter subsequently healed. In this case I had an instinctive feeling that that wisdom-tooth had something to do with this condition, and the physician was equally sure of his position that it had not.

I mention this to show that there are cases that defy the closest observation, and times when it is difficult to make a correct diagnosis of what seems to be a simple case.

In behalf of the society, I wish to thank Dr. Brown for his paper this evening, and, while regretting the absence of those men who were to have discussed the paper, we perhaps have had a greater advantage in the opportunity of questioning him than had the paper been discussed by outside parties not so interested in the dental aspect.

A vote of thanks was extended to Dr. Brown.

Adjourned.

FRED. L. BOGUE, M.D., D.D.S.,
*Editor The New York Institute of Stomatology.*

